# Disrupted Information Flow in Resting-State in Adolescents With Sports Related Concussion

**DOI:** 10.3389/fnhum.2019.00419

**Published:** 2019-12-12

**Authors:** Dionissios T. Hristopulos, Arif Babul, Shazia'Ayn Babul, Leyla R. Brucar, Naznin Virji-Babul

**Affiliations:** ^1^Telecommunication Systems Research Institute, Technical University of Crete, Chania, Greece; ^2^School of Mineral Resources Engineering, Technical University of Crete, Chania, Greece; ^3^Department of Physics and Astronomy, University of Victoria, Victoria, BC, Canada; ^4^Rockefeller College, Princeton University, Princeton, NJ, United States; ^5^Djavad Mowafaghian Centre for Brain Health, University of British Columbia, Vancouver, BC, Canada; ^6^Department of Physical Therapy, Faculty of Medicine, University of British Columbia, Vancouver, BC, Canada

**Keywords:** effective connectivity, concussion, mild traumatic brain injury, adolescents, EEG, resting state, information flow rate

## Abstract

Children and youths are at a greater risk of concussions than adults, and once injured, take longer to recover. A key feature of concussion is an increase in functional connectivity, yet it remains unclear how changes in functional connectivity relate to the patterns of information flow within resting state networks following concussion and how these relate to brain function. We applied a data-driven measure of directed effective brain connectivity to compare the patterns of information flow in healthy adolescents and adolescents with subacute concussion during the resting state condition. Data from 32 healthy adolescents (mean age =16 years) and 21 concussed adolescents (mean age = 15 years) within 1 week of injury were included in the study. Five minutes of resting state data EEG were collected while participants sat quietly with their eyes closed. We applied the information flow rate to measure the transfer of information between the EEG time series of each individual at different source locations, and therefore between different brain regions. Based on the ensemble means of the magnitude of normalized information flow rate, our analysis shows that the dominant nexus of information flow in healthy adolescents is primarily left lateralized and anterior-centric, characterized by strong bidirectional information exchange between the frontal regions, and between the frontal and the central/temporal regions. In contrast, adolescents with concussion show distinct differences in information flow marked by a more left-right symmetrical, albeit still primarily anterior-centric, pattern of connections, diminished activity along the central-parietal midline axis, and the emergence of inter-hemispheric connections between the left and right frontal and the left and right temporal regions of the brain. We also find that the statistical distribution of the normalized information flow rates in each group (control and concussed) is significantly different. This paper is the first to describe the characteristics of the source space information flow and the effective connectivity patterns between brain regions in healthy adolescents in juxtaposition with the altered spatial pattern of information flow in adolescents with concussion, statistically quantifying the differences in the distribution of the information flow rate between the two populations. We hypothesize that the observed changes in information flow in the concussed group indicate functional reorganization of resting state networks in response to brain injury.

## 1. Introduction

Traumatic brain injury (TBI) is a global health problem. In 2016 there were 27 million new cases of TBI worldwide (James et al., 2019). Mild TBI (mTBI), often used interchangeably with concussion, makes up 80–90% of all TBIs (Levin and Diaz-Arrastia, 2015). Children and youth are disproportionately affected by sport-related concussions (Moore et al., 2018) and in the last decade, there has been a significant increase in the rates of concussions in the pediatric population (Coronado et al., 2015; Taylor et al., 2015; Keays et al., 2018). Additionally, there is accumulating evidence that children and youths, once injured, take longer to recover (Barlow et al., 2010; Toledo et al., 2012). This is partly due to the fact that the effects of brain injury are overlaid on a developing brain that is undergoing dynamic changes. In addition, a concussion is itself a dynamic event characterized by spatially diffused and continually evolving secondary changes in both the brain structure and brain function.

Diffusion tensor imaging (DTI) can detect changes in the white matter microstructure of the brain. DTI shows that the stretching and tearing of the brain tissue, caused by the acceleration and deceleration forces acting upon the head during impact, result in a diffuse disconnection pattern that affects the white matter architecture of the brain. Several white matter tracks have been implicated in child and youth concussions including the corona radiata, the genu of the corpus callosum, the fornix and the cingulum, the corticospinal tract, the internal capsule, and the superior longitudinal fasciculus (Borich et al., 2013; Virji-Babul et al., 2013; Yallampalli et al., 2013; Yuan et al., 2015; Manning et al., 2017; Murdaugh et al., 2018; Wu et al., 2018). Structural damage to these tracts is due to trauma related changes in axonal membranes, myelin, intra and extra axonal edema (swelling), and inflammatory processes–see Mayer et al. (2018) for a review.

Damage to white matter pathways and traumatic axonal injuries are known to disrupt information flow across brain areas (Caeyenberghs et al., 2017). Probing the brain of a concussed youth during the “resting state” reveals significant alterations in the functional organization of the brain. The resting state of the brain is characterized by synchronous neural activity over spatially distributed networks. Our group (Borich et al., 2015) was the first to show that functional connectivity, which refers to the statistical interdependencies between the physiological time series recorded from the brain (Friston, 2011), was altered within three resting-state networks in adolescents with a concussion. Specifically, we noted: (a) alterations within the default mode network; (b) increased connectivity in the right frontal pole in the executive function network; and (c) increased functional connectivity in the left frontal operculum cortex associated with the ventral attention network (Borich et al., 2015). Newsome et al. (2016) found that asymptomatic adolescent athletes demonstrated increased connectivity (relative to a cohort of high school athletes with orthopedic injuries) between the posterior cingulate cortex and the ventral lateral prefrontal cortex, as well as between the right lateral parietal cortex and the lateral temporal cortex. More recently, Manning et al. (2017) reported significant increases in resting state connectivity in the visual and cerebellar networks.

Although individual studies show mTBI-induced alterations in different brain networks, a key feature in the above studies is an overall increase in functional connectivity, referred to as *hyperconnectivity*. Hillary et al. (2014, 2015) hypothesize that both focal and diffuse injuries associated with brain injury have widespread consequences, including the physical disruption of the neural networks and the information flow between brain regions, with hyperconnectivity being the primary response to these disruptions. The altered information flow and the hyperconnectivity can both be probed via effective connectivity (EC), which provides a measure of the influence (direct or indirect) that one brain region exerts over another (Friston, 2011) and identifies causal, directionally dependent interactions between different brain regions. However, to date, *little is known about how information flow between brain regions is actually altered following concussion*, particularly during the dynamic period of adolescent development. In this paper we investigate this issue using a data-driven effective brain connectivity measure based on the concept of information flow rate applied to EEG signals (Hristopulos et al., 2019).

The information flow rate was developed by Liang using the concept of information entropy and the theory of dynamical systems (Liang and Kleeman, 2005; Liang, 2008, 2013, 2014, 2015). Information entropy is a measure of the information contained in a given signal (e.g., time series). The theory of dynamical systems is a general framework that describes the temporal evolution of a collection of different units that can interact nonlinearly with each other while also being subject to stochastic noise signals. In studies of brain function, the individual units correspond to different brain regions or source locations, and their temporal evolution is described by means of time-ordered measurements (i.e., time series) of some property. In the present study, we focus on time-varying electrical brain activity. The information flow rate quantifies the changes in the information content of a time series (and hence, the temporal evolution of the brain region from which the signal is acquired) as a result of the interactions with other brain regions and the stochastic forces.

The information flow rate can measure the *directional transfer* of information between time series at different locations and thus between different brain regions. A high information rate from region A to region B implies that a large amount of information is transferred from A to B per second. We emphasize that a high information flow rate from A to B does not imply that the information flow rate from B to A is also high. Given a collection of source locations in the brain, the information flow rate can identify which sources transmit and which receive information, thus leading to a network of brain connections. Since the derived connectivity is directional, the information flow rate provides a method for detecting causal links in the brain.

Unlike the commonly used empirical measures of causality, e.g., transfer entropy and Granger causality, the information flow rate is derived from general, first-principles equations that describe the time evolution of stochastic dynamical systems (Liang, 2016, 2018). Additionally, owing to its definition, which involves only the time series and their temporal derivatives (or their finite-difference approximations for discretely sampled systems), the information flow rate has computational advantages over other entropy-based measures (e.g., transfer entropy), that require the estimation of additional information (e.g., conditional probabilities) from the data. In addition, the information flow rate can also be applied to deterministic nonlinear systems (Liang, 2016) without requiring a specific model structure or requiring that the time series is stationary. In terms of working with EEG signals, this endows the information flow rate analysis with a significant advantage, particularly since (a) EEG signals exhibit non-stationary features, as evidenced in transitions between quasi-stationary periods and nonlinear dynamic behavior (Blanco et al., 1995; Kaplan et al., 2005; Klonowski, 2009), and (b) the underlying model structure describing interactions between the EEG time series from different brain regions is not known. The need to assume stationarity and an underlying model structure are necessary pre-requisites for, and limitations of, other causality methods (Liang, 2015).

The aim of this study is to use the information flow rate indicated by the EEG data to measure the effective connectivity in healthy adolescents and adolescents with subacute concussions during the resting state condition and to compare the corresponding information flow rate statistics and the spatial patterns of information flow.

## 2. Materials and Methods

### 2.1. Data Collection and Pre-processing

#### 2.1.1. Participants

Thirty-two (32) right-handed, healthy, adolescent athletes (HC) [mean age: 16 years; standard deviation (SD): ±1.2] and twenty-one (21) concussed adolescents (C) (mean age = 15 years; SD: ±2.1) within one week of injury, who met the concussion diagnostic criteria consistent with the Berlin consensus statement (McCrory et al., 2017), took part in this study. Sport-related concussion is defined as a traumatic brain injury caused either by a direct blow to the head, face, neck, or elsewhere in the body with an impulsive force transmitted to the head resulting in changes in one or more of the following clinical domains: (a) somatic (such as headache), cognitive (such as brain fog), and/or emotional (such as lability) symptoms; (b) physical signs (such as loss of consciousness, amnesia, or neurological deficit); (c) balance impairment; (d) behavioral changes; (e) cognitive impairment; and (f) sleep/wake disturbances. The team coach documented the date and time of the direct blow as per the consensus statement. The team physician or an experienced physician with expertise in concussions made the diagnosis of concussion based on the Berlin consensus statement. Exclusion criteria for all individuals included focal neurologic deficits, pathology and/or those on prescription medications for neurological or psychiatric conditions.

The number of symptoms and symptom severity of each of the concussed subjects were evaluated using either the Sports Concussion Assessment Tool 3 (SCAT3), if the injured athlete was 13 years of age or older (https://bjsm.bmj.com/content/bjsports/47/5/259.full.pdf), or the Child Sports Concussion Assessment Tool 3 (Child SCAT3), if younger (https://bjsm.bmj.com/content/bjsports/47/5/263.full.pdf). Both SCAT3 and Child SCAT3 are standardized concussion and concussion symptom assessment tools. SCAT3's symptom evaluation section lists 22 symptoms that can be rated from 0 (none) to 6 (severe) while Child SCAT3 lists 20 symptoms that can be rated from 0 (none) to 3 (often). The overall symptom severity score of injured athletes was calculated by adding all the symptom ratings for a maximum score of 132 (SCAT3) or 60 (Child SCAT3).

This study was approved by the University of British Columbia Clinical Research Ethics Board (Approval number: H17-02973). The adolescents' parents gave written informed consent for their children's participation under the approval of the ethics committee of the University of British Columbia and in accordance with the Helsinki declaration. All participants provided assent.

#### 2.1.2. EEG Recording

Five minutes of resting state EEG data was collected while participants had their eyes closed. The experimental apparatus used comprises a 64-channel HydroCel Geodesic Sensor Net (EGI, Eugene, OR) connected to a Net Amps 300 amplifier (Virji-Babul et al., 2014). The signals were referenced to the vertex (Cz) and recorded at a sampling rate of 250 Hz. The scalp electrode impedance values were typically less than 50 kΩ. To eliminate artifacts caused by removing/attaching the cap, 750 data points were removed from the beginning and the end of each time series; this corresponds to removing data with a total duration of 6 s. The EEG time series were filtered using a band-pass filter (4–40 Hz) and a notch filter (60 Hz), as described in Porter et al. (2017) (see also Rotem-Kohavi et al., 2014, 2017), to remove signal drift and line noise. Eye blinks were identified and removed using an Independent Component Analysis (ICA) while motion artifacts were identified via visual inspection and also removed from the signal, as were channels with excessive noise. Each of the resulting EEG series used in this study involves between 67,845 and 114,304 time points.

#### 2.1.3. EEG Analysis

We used the Brain Electrical Source Analysis (BESA) Version 6.1 software (MEGIS Software GmbH, Gräfelfing, Germany) to map the cleaned sensor data to source waveforms. The voltages from the sensor channels are first interpolated, using spherical splines (Perrin et al., 1989; Scherg et al., 2002), to voltages at 81 predefined scalp locations that comprise BESA's Standard-81 Virtual 10-10 montage (BESA Wiki, 2018) and re-referenced to the average reference by subtracting the mean voltage of the 81 virtual scalp electrodes. The interpolation offers a consistent way of dealing with occasional bad channels while maintaining a common montage across all the individuals. Following this step, source waveforms are calculated for 15 pre-defined regional sources. Since resting-state activity is not localized, we used BESA's BR_Brain Regions montage, wherein the 15 sources are symmetrically distributed over the entire brain. BESA uses a linear inverse operator of the lead field matrix, comprising the topographies of the sources included in the source montage, to calculate the source waveforms (Scherg et al., 2002). The 15 pre-defined regions of the brain are the following: midline fronto-polar (FpM), frontal left (FL), frontal midline, (FM), frontal right (FR), anterior temporal left (TAL), anterior temporal right (TAR), central left (CL), central midline (CM), central right (CR), posterior temporal right (TPR), posterior temporal left (TPL), parietal left (PL), parietal midline (PM), parietal right (PR), and midline occipito-polar (OpM) areas. Composite source activity in each brain region is modeled as a single regional current source dipole (c.f. Hristopulos et al., 2019 for details) and source waveforms correspond to time series of the fifteen current dipoles. The resulting data was exported to MATLAB where the calculation and analysis of the information flow rates was performed.

### 2.2. Effective Connectivity

We investigated the patterns and statistics of effective connectivity by means of the information flow rate. A full description of the information flow rate for application in the analysis of EEG source-reconstructed signals is provided in Hristopulos et al. (2019). Below we provide a brief description of the measure, including the key concepts and equations.

#### 2.2.1. Information Flow Rate

In the following, $p_{i}^{\left(l\right)}\left(t_{n}\right)$ denotes the time series quantifying the time-varying strength (magnitude) of the current dipole moment at location *i* (where *i* = 1, …, *N*_*s*_ = 15) for the participant *l* = 1, …, *L* (where *L* is the number of individuals, i.e., *L* = 32 in the control and *L* = 21 in the concussed group), at time *t*_*n*_ = *n*Δ*t*, where *n* = 1, …, *N* is the time index and Δ*t* = 4 ms is the time step that corresponds to the 250 Hz sampling rate. For brevity we drop the participant index *l* and we write *p*_*i,n*_ = *p*_*i*_(*t*_*n*_) for the current dipole magnitude at source location *i* and time instant *t*_*n*_.

The overline denotes the sample time average for a single dipole time series of a given individual at the source location indexed by *i*, i.e., $\bar{p_{i}}=\frac{1}{N}\overset{N}{\underset{n=1}{\sum }}p_{i,n}$. The *sample cross-covariance* of two time series corresponding to dipoles *i* and *j* is respectively given by:

$$\begin{array}{l} {\hat{C}}_{i,j}=\bar{p_{i,n}p_{j,n}}-\bar{p_{i,n}}\bar{p_{j,n}},\text{ for }i,j=1,\ldots ,N_{s}. \end{array}$$

If *i* = *j*, the above equation gives the sample variance of the time series *p*_*i*_, i.e., ${\hat{\sigma }}_{i}^{2}\equiv {\hat{C}}_{i,i}$.

The linear (Pearson) sample correlation coefficient between the series *p*_*i*_ and *p*_*j*_ is defined by the ratio:

(1) $$\begin{array}{l} {\hat{r}}_{i,j}=\frac{{\hat{C}}_{i,j}}{{\hat{\sigma }}_{i}{\hat{\sigma }}_{j}},\text{ for }i,j=1,\ldots ,N_{s}. \end{array}$$

Both ${\hat{C}}_{i,j}$ and ${\hat{r}}_{i,j}$ are non-directional correlation measures. Pearson's correlation coefficient has been used as a measure of functional connectivity (Sakkalis, 2011).

We also consider the sample covariance of the time series *p*_*i*_ and the first derivative of the series *p*_*j*_, ${\hat{C}}_{i,dj}$, which we use to compute the cross-correlation coefficients, ${\hat{r}}_{i,dj}$, where *i, j* = 1, …, *N*_*s*_, between the time series *p*_*i*_ and the temporal derivative, ${\dot{p}}_{j}$, of the time series *p*_*j*_ (Liang, 2014). These coefficients are expressed in terms of the respective covariances as follows:

(2) $$\begin{array}{l} {\hat{r}}_{i,dj}=\frac{{\hat{C}}_{i,dj}}{{\hat{\sigma }}_{i}{\hat{\sigma }}_{j}},\text{ for }i,j=1,\ldots ,N_{s}. \end{array}$$

As for ${\dot{p}}_{j}$, since it is unknown *a priori*, a finite difference approximation based on the Euler forward scheme, with a time step equal to *k*Δ*t*, is used, i.e.,

(3) $$\begin{array}{l} {\dot{p}}_{j,n}=\frac{p_{j,n+k}-p_{j,n}}{k\Delta t},\text{ for }j=1,\ldots ,N_{s},\;n=1,\ldots ,N. \end{array}$$

Based on the discussion in Liang (2014) and Hristopulos et al. (2019), we use *k* = 2.

The information flow rate, which measures the rate of information transfer from the time series *i* to the time series *j*, can be expressed using the above definitions as follows (Liang, 2014):

(4) $$\begin{array}{l} T_{i\to j}=\frac{{\hat{r}}_{i,j}}{1-{\hat{r}}_{i,j}^{2}}\left({\hat{r}}_{i,dj}-{\hat{r}}_{i,j}{\hat{r}}_{j,dj}\right),\\ \text{ for }i,j=1,\ldots ,N_{s},\;i\neq j. \end{array}$$

We refer to *p*_*i*_ as the *transmitter series* and to *p*_*j*_ as the *receiver series* with respect to *T*_*i*→*j*_. A positive (negative) rate of information flow from *i* → *j* (*T*_*i*→*j*_) indicates that the interaction between the two series leads to an increase (decrease) in the entropy of the series *p*_*j*_. Equivalently, it signifies that the *receiver* series becomes more (less) unpredictable due to its interaction with the *transmitter* series. The predictability of each time series is negatively correlated with the entropy.

#### 2.2.2. Normalized Information Flow Rate

The information flow rate *T*_*i*→*j*_ is a measure of the information flow from series *p*_*i*_ to series *p*_*j*_; however, it offers no indication of whether the impact of *p*_*i*_ on the predictability of *p*_*j*_ is significant. Quantifying the latter requires knowing the *relative impact* of the entropy transferred to the *receiver* from the *transmitter* series, compared to the total entropy rate of change due to all the influences acting on the *receiver*. The latter (hereafter referred to as the normalization factor for the information flow rate from *p*_*i*_ to *p*_*j*_ and denoted as *Z*_*i*→*j*_) can be straightforwardly computed (Liang, 2015; Hristopulos et al., 2019). The relative impact of the *transmitter* series on the *receiver* series is then given by the *normalized information flow rate* from the *transmitter p*_*i*_ to the *receiver p*_*j*_:

(5) $$\begin{array}{l} \tau_{i\to j}=T_{i\to j}/Z_{i\to j}, \end{array}$$

which measures the percentage of the total entropy rate of change for *p*_*j*_ that is due to its interaction with *p*_*i*_. Thus, in the following we use τ_*i*→*j*_ to quantify the resting-state effective connectivity in the brains of the control (healthy) and concussed individuals, and to investigate the patterns of directional information flow between the different regions of their brains.

## 3. Results

In Table 1 we list the demographic information about the participants in this study. All the concussed participants met the Berlin criteria and exhibited a minimum of 4 and as many 22 SCAT3 symptoms at the time of testing. The most common symptoms were difficulty concentrating/remembering, dizziness, sensitivity to light, “don't feel right,” fatigue, and irritability.

**Table 1:** Demographic information for the participants in the control and concussed groups.

**Demographic information**	**Controls**	**Concussed**
Age (Years, SD)	16 (1.2)	15 (2.1)
Gender	100% Male	100% Male
Time since concussion		100%: within 1 week
SCAT3 (# of Symptoms, SD)		13.1 (7.0)
SCAT3 (Symptom Severity, SD)		28.8 (19.0)
Child-SCAT3 (# of Symptoms, SD)		12.5 (6.3)
Child-SCAT3 (Symptom Severity, SD)		23 (19.7)

*The acronym “SD” stands for “standard deviation” and “SCAT” for “Sports Concussion Assessment Tool”*.

**Figure 1:**
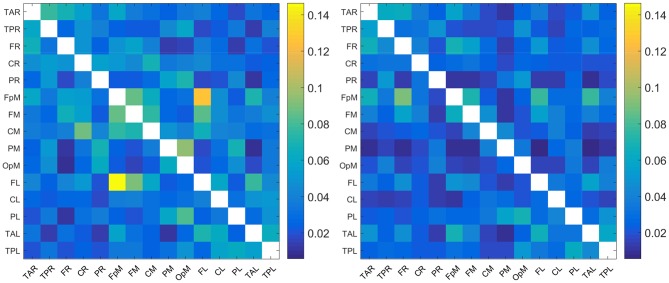
Maps of the mean absolute normalized information flow rate |*τ*_*i*→*j*_| for the control **(Left)** and concussed **(Right)** individuals. The EEG source labels are defined in Table 2.

The plots in Figure 1 show the mean of |*τ*_*i*→*j*_| evaluated over all the individuals in the control and concussed groups respectively. The acronyms appearing in the figure refer to EEG source locations in the brain listed in Table 2.

**Table 2:** List of the 15 brain regions used in EEG source space reconstruction in BESA.

**Source dipole label**	**Brain region**
FL	Frontal, left hemisphere
FpM	Fronto-polar midline
FR	Frontal, right hemisphere
FM	Frontal midline
CL	Central, left hemisphere
CM	Central midline
CR	Central, right hemisphere
TPL	Posterior temporal, left hemisphere
TAL	Anterior temporal, left hemisphere
TAR	Anterior temporal, right hemisphere
TPR	Posterior temporal, right hemisphere
PM	Parietal midline
PL	Parietal, left hemisphere
PR	Parietal, right hemisphere
OpM	Occipital-polar

Since the reconstructed EEG signal involves 15 source locations, the total number of potential connections between brain regions is 15 × 14 = 210 (the 15 self-connections *i* → *i* are not meaningful and are thus excluded). As evidenced in Figure 1, the range of values of |*τ*_*i*→*j*_| for the two groups are broadly comparable (we are using a common color scale for the two plots). In detail, however, the values of mean |*τ*_*i*→*j*_| are overall somewhat higher for the control group and the connectivity patterns are different. We elaborate on the connectivity differences below.

### 3.1. Qualitative Comparison of Information Flow Rates and Connectivity Patterns

**Figure 2:**
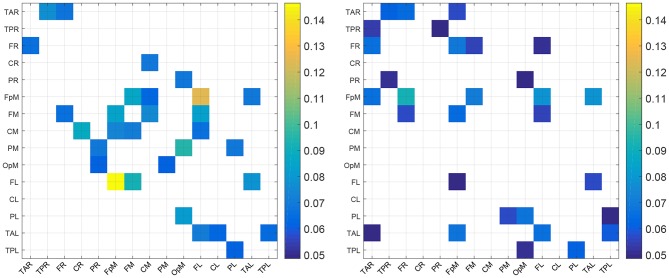
Maps of the top 30 mean absolute normalized information flow rate values for the control **(Left)** and concussed **(Right)** individuals. The EEG source labels are defined in Table 2.

**Figure 3:**
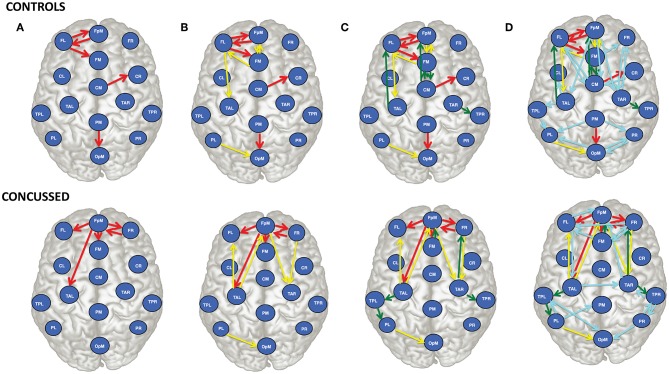
Brain view comparison of the information flow rate between healthy controls (top row) and concussed (bottom row). Schematics **(A–D)** display respectively from left to right the top 5, 10, 15, and 30 connections. The connections are ranked based on the average value of |*τ*_*i*→*j*_|. The EEG source labels are defined in Table 2.

For purposes of a qualitative comparison, we limit ourselves to the thirty (30) strongest connections (for example the connections with the largest |*τ*_*i*→*j*_|) for the control and the concussed individuals. These connections are listed in a rank-ordered fashion in Table 3 and shown in Figure 2 in terms of a matrix plot. The magnitudes of these top thirty connections range between 0.146 and 0.06 for the control group and between 0.091 and 0.049 for the concussed, and an examination of Figure 2 suggests that the spatial distribution of the two sets of connections is manifestly different. In the case of the control group, the connections are more clustered about the diagonal as well as toward the center of the matrix. In the case of the concussed group, the connections are more dispersed and there is a larger number of them near the edges. The connections are also displayed in Figures 3A–D, by means of directional arrows on the axial-view brain schematics: Figure 3A illustrates the spatial distribution of the five *most* important connections (red arrows); Figure 3B shows the top ten connections, with those ranked 6 to 10 shown in yellow. Figure 3C shows the spatial distribution of the top 15 connections, with those ranked from 11 to 15 plotted in dark green, while Figure 3D gives the spatial distribution of the full set of top thirty connections, with those ranked 15 to 30 colored turquoise.

**Table 3:** List of the thirty most active connections in source space for the *control* (left) and *concussed* (right) individuals ranked by mean |*τ*_*i*→*j*_|.

	**From**	**To**	**Mean **|*τ*_*i*→*j*_|****	**From**	**To**	**Mean **|*τ*_*i*→*j*_|****
	**Control**			**Concussed**		
1	FL	FpM	1.463516e-01	FpM	FR	9.139760e-02
2	FpM	FL	1.244544e-01	FpM	TAL	7.800451e-02
3	PM	OpM	9.544217e-02	FpM	FL	7.796168e-02
4	FL	FM	9.151434e-02	FpM	FM	7.068658e-02
5	CM	CR	8.715951e-02	FR	FpM	6.849991e-02
6	FpM	FM	8.528051e-02	PL	OpM	6.841781e-02
7	FM	FpM	8.513447e-02	TAL	FpM	6.758274e-02
8	FM	FL	8.300400e-02	FpM	TAR	6.683954e-02
9	PL	OpM	8.091458e-02	FR	TAR	6.599597e-02
10	FL	TAL	7.785959e-02	TAL	FL	6.582645e-02
11	TAR	TPR	7.631225e-02	TAR	FR	6.447614e-02
12	FM	CM	7.534613e-02	FM	FpM	6.404960e-02
13	CM	FpM	7.455397e-02	TPL	PL	6.224385e-02
14	CM	FM	7.023347e-02	TAR	TPR	6.117049e-02
15	TAL	FL	7.021745e-02	TAL	TPL	5.957048e-02
16	FpM	TAL	6.997750e-02	TAR	FpM	5.773160e-02
17	PR	OpM	6.969384e-02	PL	PM	5.766076e-02
18	PM	PR	6.927217e-02	FM	FR	5.691796e-02
19	CR	CM	6.851740e-02	FL	TAL	5.649172e-02
20	PM	PL	6.850605e-02	FR	FM	5.594106e-02
21	TAR	FR	6.783224e-02	FM	FL	5.527661e-02
22	FR	TAR	6.657403e-02	TPR	TAR	5.410975e-02
23	CM	FL	6.640744e-02	PR	TPR	5.153787e-02
24	FM	FR	6.630883e-02	TPL	OpM	5.132545e-02
25	TAL	TPL	6.473654e-02	FR	FL	5.126175e-02
26	FpM	CM	6.348101e-02	PR	OpM	4.983997e-02
27	TAL	CL	6.266565e-02	FL	FpM	4.933237e-02
28	TPL	PL	6.158443e-02	PL	TPL	4.897207e-02
29	OpM	PR	6.095310e-02	TAL	TAR	4.892466e-02
30	OpM	PM	6.082626e-02	TPR	PR	4.862880e-02

*The EEG source labels are defined in Table 2*.

An examination of Figure 3A reveals that of the top 5 connections for the *control group*, three involve the left frontal region (FL): FL sends information to the frontal midline and fronto-polar midline (FM, FpM), and receives information from FpM. The bidirectional connection is on the left side. In the *concussed group*, however, the most active site is the fronto-polar midline region (FpM), with the top four connections extending bilaterally to the left and right frontal regions (FL, FR) as well as to frontal midline (FM) and left temporal (TAL) region. The one bidirectional connection is on the right side, between FpM and FR.

Introducing the next five connections (6 to 10) and examining the top ten connections jointly (Figure 3B), we find that in the *control group*, the connections are largely concentrated in the left anterior region of the brain. We also note that there is now a total of three bidirectional connections: between left frontal (FL) and fronto-polar midline (FpM) that we have already highlighted as well as between left frontal (FL) and frontal midline (FM) and between fronto-polar midline (FpM)and frontal midline (FM) regions.

In contrast, in the *concussed group*, the left-right symmetry in the distribution of the connections becomes more apparent; the activity extends beyond the frontal regions and involves both the left and right temporal regions (TAL and TAR). We also observe two pairs of bidirectional connections: between FpM and FR already mentioned as well as between FpM and TAL.

For the *control group*, the next five connections (11 to 15) largely preserve the existing spatial distribution of the connections. The flow is still primarily left lateralized and anterior-centric. The additional connections do expose additional bidirectional information flow between already engaged brain regions. In the *concussed* data, the left-right symmetry in the distribution of the connections in the anterior region of the brain is largely unaltered; however, we also observe the information flow extending toward the posterior regions of the brain, with a slight excess in the number of such connections on the left.

With the final fifteen to thirty connections (in Figure 3D), we see the emergence of distributed activity in the posterior half of the brain in both the *control* and *concussed groups*. This includes bidirectional flows between the parietal and the occipital regions in the *control group*, and the temporal and the parietal regions in the *concussed groups*. Additionally, in both groups, the additional connections tend, on the whole, toward greater left-right symmetry in both the posterior as well as the anterior regions. The *concussed* results also show the emergence of two inter-hemispheric connections between the left and right temporal regions (TAL to TAR) and the left and right frontal regions (i.e., FR to FL).

### 3.2. Statistical Comparisons of Information Flow Rates and Connectivity Patterns

First, we have assessed the top thirty connections discussed above and have confirmed the statistical significance of these top |*τ*_*i*→*j*_| values using the *non-parametric permutation testing* (Maris and Oostenveld, 2007; Cohen, 2014). In permutation testing, we formulate a *null hypothesis* that there is no information flow between the sources *p*_*i*_ and *p*_*j*_ for all *i* and *j*. We then generate *M*_*s*_ randomized states *u*_*m*_(*t*_*n*_), where *n* = 1, …, *N*, and *m* = 1, …, *M*_*s*_. The randomized states are derived from each transmitter source time series *p*_*i*_ by scrambling the *N* time points of *p*_*i*_ using random permutations. This operation destroys the temporal order of *p*_*i*_ and also any patterns of information flow from *p*_*i*_ to *p*_*j*_. Any resulting deviations of the estimated |*τ*_*m*→*j*_| values (based on the shuffled time series *p*_*m*_) from zero, are due to random fluctuations and do not represent meaningful information flow. Statistically significant values of information flow should be considerably higher than the random fluctuations of |*τ*_*m*→*j*_|.

The *p*-value of the statistic |*τ*_*i*→*j*_| is defined as the fraction of the *M*_*s*_ permutation states for which |*τ*_*m*→*j*_| exceeds |*τ*_*i*→*j*_|. A high *p*-value implies that the null hypothesis cannot be rejected since a significant number of randomized states have higher information flow rate than the |*τ*_*i*→*j*_|. In contrast, a low *p*-value provides support for the alternative hypothesis (i.e., statistically significant information flow from *p*_*i*_ to *p*_*j*_). The observed value |*τ*_*i*→*j*_| is then considered as statistically significant if the respective *p*-value is below a specified significance level. We use 0.05 for the latter for all the statistical tests reported below although we also report the actual *p*-value as well.

In our numerical analysis, we used *M*_*s*_ = 100. For each simulation and each individual we scramble the time order of the transmitting source *p*_*i*_ for all the top 30 transmitter-receiver pairs *p*_*i*_ → *p*_*j*_, and we calculate the resulting |*τ*_*m*→*j*_|. For the (control, concussed) group, we thus have a matrix of (32, 21) (individuals) ×30 (top connections) ×100 (# simulations) values of |*τ*_*m*→*j*_|. We take the average over the individuals within each group leading to two 30 × 100 matrices of |*τ*_*m*→*j*_| values. Then, we count how many of the 100 average |*τ*_*m*→*j*_| values (based on the randomized transmitter series) exceed the group's mean |*τ*_*i*→*j*_| for each of the top 30 connections in each group. The fraction of such exceedances gives us the *p*-value. For both the control and the concussed groups the resulting *p*-value is zero. In the control (concussed) group the lowest average |*τ*_*m*→*j*_| is ≈ .6 × 10^−6^ (4.98 × 10^−6^) and the highest is ≈ .3.53 × 10^−5^ (4.26 × 10^−5^). These values are well below the range of the |*τ*_*i*→*j*_| values for the top 30 connections for both groups reported in Table 3. It is also possible to obtain *p*-values by calculating standard *Z* values for the observed |*τ*_*i*→*j*_| in each group based on the null hypothesis distribution (Cohen, 2014, p. 464). This approach rests on the assumption of a normal distribution of the group averages. It is somewhat more conservative, since it can yield non-zero *p*-values even if the approach based on the fraction of exceedances yields a zero *p*-value. However, in the present case, the standard *Z*-value approach also leads to zero *p*-values. Hence, since our analysis is based on 100 randomized samples, we can conclude that the top thirty within-group-averaged |*τ*_*i*→*j*_| values are statistically significant at the 5% level for both groups.

**Figure 4:**
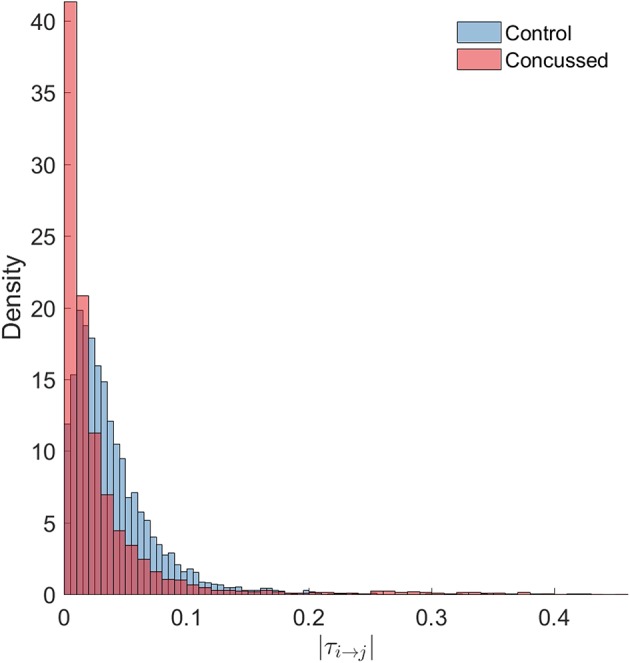
Probability density histograms of the |*τ*_*i*→*j*_| values for the control (blue-gray) and concussed (rose) groups. The height of each histogram bar is equal to the number of observations in the bin divided by the total number of observations and the bin width. Thus, the area of each bar is equal to the relative number of observations per bin, and the sum of all the bar areas is equal to one.

Next, we look at *all* the connections and perform statistical comparisons of the normalized information flow rates obtained for the groups of control and concussed individuals. The question that we seek to answer is whether the marginal distributions of information flow rate are different in these two groups, and whether such difference, if it exists, leads to distinguishable summary statistics. The following analysis of the |*τ*_*i*→*j*_| empirical (sampling) distributions is based on the 32 × 210 = 6720 values for the control and the 24 × 210 = 5040 values for the concussed group. The |*τ*_*i*→*j*_| values are calculated over all the individuals in each group and for each of the 210 pairs of 15 source locations (as discussed in section 3.1) and the two distributions are shown in Figure 4.

We test the null hypothesis that the |*τ*_*i*→*j*_| for both the control and concussed groups follow the same marginal (1-D) statistical distribution. We use the *Kolmogorov-Smirnov (K-S)* test for comparing the two corresponding cumulative distribution functions, e.g., Press et al. (2007). The null hypothesis is that there is no difference between the two distributions (for the control and concussed groups). The K-S test rejects the null hypothesis with *p* = 1.6175 × 10^−214^, which is practically zero. Moreover, unlike the histograms shown in Figure 4, the K-S test operates on the raw data and is not affected by the bin size. This result provides solid statistical evidence that the probability distributions of the |*τ*_*i*→*j*_| values for the control and concussed groups are different. This result is also supported by the probability density histograms shown in Figure 4: in the concussed group there is a higher probability for smaller values of |*τ*_*i*→*j*_| than in the control group, as well as a longer right tail which implies the presence of some higher values in the concussed group.

We also use the non-parametric Kruskal-Wallis test (Durka et al., 2004) to compare the |*τ*_*i*→*j*_| distributions for the control and concussed groups. The data are assumed to come from continuous distributions which are otherwise arbitrary. The null hypothesis is that two samples come from the same distribution. The Kruskal-Wallis test is a non-parametric one-way analysis of variance (ANOVA) which tests the null hypothesis. In contrast with the classical parametric ANOVA method, the Kruskal-Wallis test does not rely on the normal distribution.

Since the control group involves 32 subjects and the concussed group 21, we perform the comparison between the two groups using 1,000 samples. Each sample involves 210 × 21 |*τ*_*i*→*j*_| values obtained from 21 out of 32 control individuals and the respective |*τ*_*i*→*j*_| values for the 21 concussed individuals. In all cases the Kruskal-Wallis test shows that the null hypothesis is rejected with *p* < 1.95 × 10^−131^. Hence, this test also confirms that the |*τ*_*i*→*j*_| values from the two groups do not follow the same probability distributions.

The descriptive *summary statistics* for the |*τ*_*i*→*j*_| sampling distributions of the two groups are given in Table 4. We compare the median value, the coefficient of variation (COV) which is equal to the standard deviation divided by the mean, the skewness (coefficient of asymmetry), and the kurtosis coefficient. For the COV, the skewness and the kurtosis coefficients we provide estimates of uncertainty (95.45% confidence intervals) that are based on two times the *jackknife estimate* of standard error (Efron and Hastie, 2016, p. 156). The jackknife estimates are obtained by generating *L* samples (*L* being the number of individuals in each group), so that each sample excludes one of the individuals in the group. The jackknife is known to overestimate the true standard error (Efron and Hastie, 2016, p. 158). The respective 95.45% confidence intervals for the summary statistics of the two groups are non-overlapping. This result supports the conclusion of the K-S test, i.e., that the probability distributions of the |*τ*_*i*→*j*_| values for the control and concussed groups are different.

**Table 4:** Statistics of the |*τ*_*i*→*j*_| sampling distributions from the individuals in the control and concussed groups.

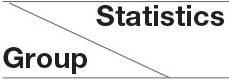	**Median**	**COV**	**Skewness**	**Kurtosis**
Control	0.030	0.89 ± 0.066	2.30 ± 0.44	12.07 ± 5.35
Concussed	0.013	1.77 ± 0.13	4.15 ± 0.41	23.24 ± 4.22

*COV is the coefficient of variation. The intervals reported for the COV, the skewness and the kurtosis coefficients are equal to two times the jackknife estimate of the standard error*.

**Figure 5:**
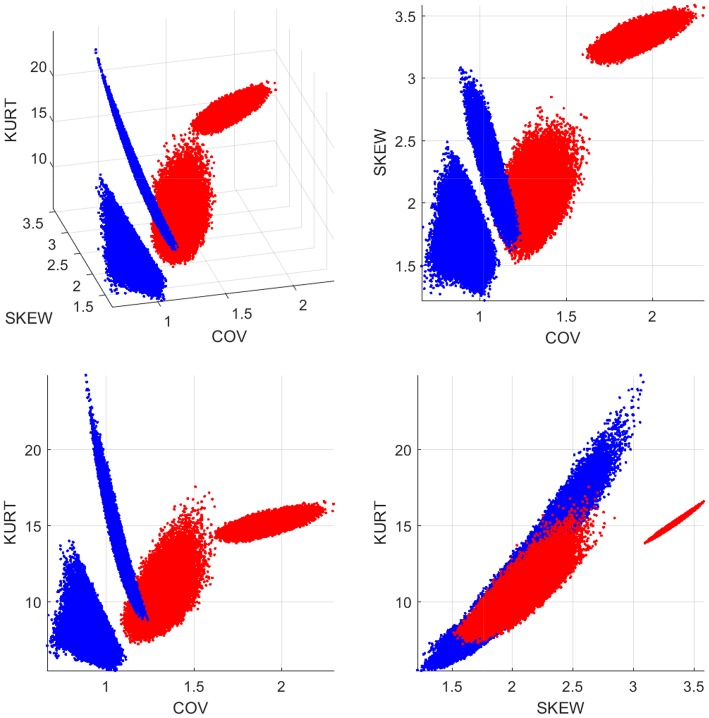
Statistics of the |*τ*_*i*→*j*_| distributions for the control (blue dots) and concussed (red dots) groups. The coordinates of each point in 3D space comprise the coefficient of variation (COV), the coefficient of asymmetry (SKEW), and the kurtosis coefficient (KURT). The 100 000 points are generated by random sub-sampling of 12 individuals from each group.

To further investigate the differences between the two |*τ*_*i*→*j*_| empirical distributions, we generate 100,000 sub-samples comprising 12 individuals for each group. The sub-samples per group are generated by means of random permutations of the individuals' indices. The coefficient of variation, the skewness and the kurtosis of |*τ*_*i*→*j*_| are evaluated for each sub-sample (based on all the connections and individuals in the sub-sample) and the results are plotted in Figure 5. The four plots shown include the 3D scatter plot of the three-component statistical vector (COV, skewness, kurtosis) (top left) as well as the three 2D projections on the planes (COV, skewness) [top right] (COV, kurtosis) [bottom left] and (skewness, kurtosis) [bottom right]. As it is evident in these plots, there is a clear separation between the |*τ*_*i*→*j*_| “points” in the control and concussed groups. In addition, the patterns formed by the statistics viewed as points in 3D space have different geometric shapes and orientations.

The final important question that we consider is whether the observed difference in the spatial organization of the effective connectivity between the control and concussed groups is statistically significant. The qualitative survey of the connectivity patterns manifested by the top 30 connections in the two groups hint at significant differences. However, we would like to quantify this. To that end, we investigate this question in terms of a summary statistic that reflects the character of the information flow connections between the fifteen brain regions (i.e., source locations). Specifically, we construct weighted, asymmetric networks (graphs) based on the τ_*i*→*j*_ values of each group, and then compare the resulting network patterns in terms of a characteristic measure of the network's topological structure. The selected measure is the *assortativity coefficient* introduced by Newman (2002) and Noldus and Van Mieghem (2015). The calculation involves the following steps:

1. The source locations correspond to the nodes of the network and the edges correspond to connections.
2. We construct the *network adjacency matrix A*_*i,j*_ = |*τ*_*i*→*j*_| for all possible connections between source locations. The matrix **A** has positive values (weights) for all the connected edges and zero otherwise.
3. Each node is characterized by two numbers: the in-degree (i.e., the weighted sum of the incoming connections), and the out-degree (i.e., the weighted sum of the outgoing connections).
4. We characterize the network connectivity in terms of the *degree assortativity coefficient* for weighted, directed networks, e.g., Rubinov and Sporns (2010) using the function pearsonW.m in the Octave networks toolbox (Bounova, 2014). The degree assortativity coefficient, *r*_*W*_, of a network is the Pearson correlation coefficient between strengths (weighted degrees) of all nodes at the opposite ends of an edge. It is a measure for the tendency of nodes to connect with other nodes that have similar strength. It is an important global measure of a network's topological structure and is frequently used in brain network analysis, e.g., Rubinov and Sporns (2010); Geier et al. (2015); Betzel et al. (2018) and Lim et al. (2019).
5. We formulate the null hypothesis that there is no statistically significant difference in *r*_*W*_ of the two groups. We then use permutation testing to test this hypothesis (Cohen, 2014, p. 461). If there is no difference between the control and concussed individuals (i.e., if the null hypothesis is valid), the values of the assortativity coefficient computed for the individuals in the control and concussed groups should be contained within the respective null hypothesis distributions.
6. We combine the information flow rates for both the control and the concussed individuals into a single set reflecting the null hypothesis.
7. We randomly partition this set into two subsets, one containing 32 subjects (used to test the null hypothesis in the control group) and one containing 24 subjects (to test the null hypothesis in the concussed group).
8. We calculate the mean absolute information flow rate for all the individuals in each group (partition). We then compute *r*_*W*_ based on these averages.
9. We repeat the random partitioning for *N* ≫ 1 times (e.g., *N* = 10, 000), generating *N* values of *r*_*W*_ and in the process, obtaining a probability distribution of *r*_*W*_ for each group.
10. We compute the percentage of times that *r*_*W*_ values obtained from the null hypothesis distribution are more extreme than the respective values obtained for the two separate groups; this percentage gives the respective *p* value.

**Figure 6:**
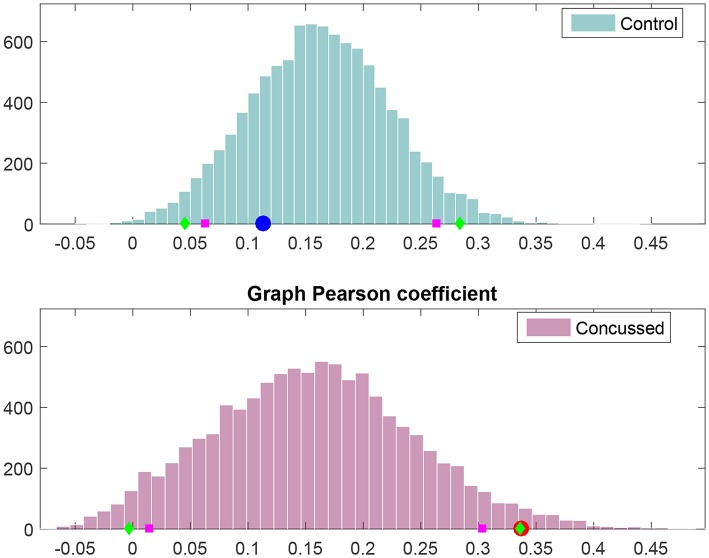
Frequency histograms of Pearson's degree-degree correlation coefficient under the null hypothesis (i.e., no difference between the control and concussed groups). The top histogram is for the control group and the bottom histogram for the concussed group. The 5 and 95% percentiles are marked by magenta squares, while the 2.5 and 97.5% percentiles are marked by green diamonds. The value obtained for the control group is marked by the blue circle **(Top)**, and the value obtained for the concussed group is marked by the red circle **(Bottom)**.

The results of the analysis outlined above are displayed in Figure 6. It is evident that *r*_*W*_ for the control group individuals is explained by the null hypothesis for the control group. However, *r*_*W*_ for the concussed group corresponds to *p* = 0.025 with respect to the corresponding null hypothesis distribution; thus, the null hypothesis can be rejected at the 5% level.

To summarize, we have carried out a quantitative statistical analysis of the *marginal probability distributions* of the individual |*τ*_*i*→*j*_| as well as the group *effective connectivity patterns*, based on the mean |*τ*_*i*→*j*_|. The results demonstrate that not only is the spatial organization of the effective connectivity of the control and concussed groups different, the individual information flow rates in each group also follow distinct marginal probability distributions.

## 4. Discussion

In this study we investigated the changes in effective connectivity between a group of adolescent athletes with subacute sports related concussion and a group of adolescent athletes with no previous history of concussion. We applied the information flow rate to the EEG time series signals from different source locations to measure the transfer of information between different brain regions. Based on the ensemble means of the normalized information flow rate, our analysis revealed acute changes in effective connectivity compared with age-matched controls.

We find that the strongest information flows in adolescent athletes with no previous history of concussion is primarily left (L) lateralized and anterior-centric, and dominated by the triangular nexus of bidirectional information exchange between brain regions of comparable degree: FL, FpM, FM, and CM. (Degree refers to the total number of incoming flows and outgoing connections). The connections in the right anterior and in the posterior regions are typically weaker. The latter are more left-right (L-R) symmetric, with both the left and right parietal regions engaging in bidirectional information exchange with the occipital region.

In contrast, adolescents with subacute concussion show four distinct changes in the pattern of information flow during resting state. First, instead of four regions of comparable degree, one region, the fronto-polar midline (FpM), stands out above all others. Second, while the strong information flow connections are still concentrated in the anterior region of the brain, their spatial distribution is much more (L-R) symmetrical and include strong bidirectional connections between the frontal and the temporal (TAL and TAR) regions. Third, we observe the presence of two inter-hemispheric connections in the anterior region, i.e., FR → FL and TAL → TAR. The inter-hemispheric connections are not present in the control group. Lastly, the activity in the central and the parietal midline regions is greatly reduced, and the centers of activity in the posterior regions of the brain has shifted from the parietal regions to the left and right posterior temporal regions, with the bidirectional information exchange now occurring between the posterior temporal and the parietal regions on both the left and the right. Below we consider these results with the context of the functional hyperconnectivity hypothesis.

### 4.1. Hyperconnectivity as a Feature of Brain Injury

Hillary and Grafman (2017) argues that hyperconnectivity (as measured by an increase in the magnitude or the number of connections in brain regions), is a fundamental and observable response to all neurological injury resulting in neural network disruption. They propose that this response reflects the brain's attempt to re-establish communication between networks through the recruitment of “detour pathways” using less established routes to bypass prior, now damaged, connections. While a growing number of studies demonstrate hyperconnectivity following concussion (Borich et al., 2015; Newsome et al., 2016; Manning et al., 2017), these studies tend to focus on increases in the density of connections. To date there is no study explicitly demonstrating the formation of re-routing patterns via an effective connectivity analysis. Our results are the first to show this. Specifically, our analysis demonstrates the emergence of four alternate pathways of information flow in the concussed group that likely reflect the consequences of physical disruption of prior connections.

First, we observed a shift in the center of activity from the left frontal region to the fronto-polar midline region, and a concomitant increase in the number of strong information flow connections in the right anterior region that results in a more (L-R) symmetrical connectivity pattern. These changes are consistent with results of our previous resting state fMRI study and resting EEG study of functional connectivity in concussed versus healthy (control) subjects. In the fMRI study, we found that increased functional connectivity was primarily concentrated in the right frontal region within the executive function network (Borich et al., 2015). The EEG study showed significant increases in the functional connectivity in areas corresponding to the right inferior frontal gyrus and the right dorsolateral prefrontal cortex (Virji-Babul et al., 2014), areas that in Figure 3 lie to the right of FM. Numerous studies have reported a frontal and specifically, prefrontal vulnerability to brain injury —see Eierud et al. (2014) for review. In adolescence, the incomplete development of white matter tracts may contribute to the increased vulnerability in this region. Damage to these areas (both left and right) may require re-routing of information via newly established detour pathways to re-establish communication within frontal-central regions.

The next three key findings in the concussed group, which we consider together, are (a) the presence of two inter-hemispheric connections, one in the frontal region, i.e., FR→ FL, and one between the left and right temporal regions, i.e., TAL → TAR, (b) a shift to bilateral posterior temporal regions, and (c) the diminished activity in the central and the parietal midline regions, and to a lesser degree in the frontal midline region.

These shifts in information flow are most likely a consequence of injury to white matter tracts such as the corona radiata, the genu of the corpus callosum and the superior longitudinal fasciculus. In fact, Ling et al. (2012) have suggested that the fibers within the corpus callosum are highly susceptible to mechanical injury and several studies have shown that concussion results in impact to the corpus callosum (Kraus et al., 2007; McAllister et al., 2012). We hypothesize that injury to the interface between the two hemispheres of the brain, and to the white matter tracts, particularly the corpus callosum, is the reason for the reduced activity in the frontal-central-parietal midline regions and the emergence of these alternate pathways of information flow between the two hemispheres that we have observed in this study.

An important question that arises from these results is: What are the functional consequences of hyperconnectivity and the establishment of alternate pathways of information flow? It is important to note that hyperconnectivity does not necessarily represent positive functional plasticity. Caeyenberghs et al. (2017) have proposed that increased functional connectivity may represent maladaptive plasticity, particularly when associated with impaired cognitive function. Increased functional connectivity is metabolically costly and may be associated with a highly simplified and tightly synchronized pattern of brain activation that constrains the dynamics and flexibility of neural responses (Hillary et al., 2015; Caeyenberghs et al., 2017). Although the behavioral correlates of this type of plasticity or re-structuring have not been well characterized, this altered functional connectivity may limit the performance of tasks that require dynamic switching between different brain/behavioral states.

We recently investigated how pediatric concussion alters the temporal dynamics of brain states within resting state networks using resting state fMRI data. Functional networks in resting state are not stationary but rather switch between different brain states. The strength as well as the direction of connections vary from seconds to minutes (Chang and Glover, 2010; Jones et al., 2012; Hutchison et al., 2013). Using a sliding window analysis, we extracted three separate brain states within the resting state condition in both healthy adolescents and adolescents with concussion. Our analysis revealed that the healthy adolescents switched dynamically between three brain states, spending approximately the same time in each brain state. In contrast, we found that adolescents with concussion spent the majority of time in only one brain state. We hypothesize that this lack of dynamic flexibility is likely to negatively impact the performance of tasks that require shifting of attentional states or performance of more complex tasks (Muller and Virji-Babul, 2018).

One potential long-term concern for concussed patients is that this pattern of hyperconnectivity, combined with limited flexibility of network dynamics, may represent more than a transient process of brain injury. Since hyperconnectivity is associated with high metabolic cost, chronic hyperconnectivity, in combination with elevated metabolic processes may offer a clue to the link between concussion and future neurodegeneration. Clearly, long-term studies examining the trajectory of connectivity and the relationship between structural, functional and effective connectivity patterns are urgently needed—particularly in the adolescent phase of development.

### 4.2. Limitations

There are several limitations to this study that are worth noting. First, we have a modest sample size which comprises data obtained at one time point between 1 week and 1 month post injury. Thus, the effects that we report will likely continue to evolve over the course of recovery. Longitudinal studies over the span of at least 6 months to 1 year are needed to understand the long-term effects on effective connectivity resulting from a single concussion. Several important questions need to be answered, such as: how does recovery of white matter integrity influence the pattern of effective connectivity, what is the relationship and trajectory of both brain structure and function over a 1 year time span in the adolescent brain, and how do these changes impact high level cognitive function? The second limitation is that our sample primarily consists of male adolescent athletes. This constraint is due to the sports teams that agreed to participate in our study. The effects of concussion on female athletes are highly understudied. We are currently collecting data and following a group of female soccer players over an entire season to evaluate the effects on brain connectivity in this group.

In summary, our study demonstrates the changes in effective connectivity associated with a sports related concussion (within 1 week of injury) in an adolescent population, for the first time. The acute effects of a concussion are shown to be associated with distinct differences in information flow in comparison with age-matched youths who had no history of concussion. Specifically, the concussed group shows a more left-right symmetric pattern of information flow corresponding to an increase in the number of strong connections in the right anterior region of the brain, inter-hemispheric connections between the left and right frontal and temporal regions of the brain, and a diminished level of activity along the frontal-central-parietal midline axis. They also highlight the need to follow athletes longitudinally in order to study the long-term impact of concussion, particularly when the concussion occurs during the dynamic period of adolescent brain development.

## Data Availability Statement

The datasets generated for this study are available on request to the corresponding author.

## Ethics Statement

The studies involving human participants were reviewed and approved by University of British Columbia Clinical Research Ethics Board (Approval number: H17-02973). Written informed consent to participate in this study was provided by the participants' legal guardian/next of kin.

## Author Contributions

DH contributed to the analysis of the data, the methodological aspects of the study, and the writing of the manuscript. AB was involved with the methodological development, the conceptual analysis of the results, and the writing of the manuscript. SB worked on the analysis of the data. LB worked on the preparation and pre-processing of the data and contributed to the writing of the manuscript. NV-B designed the study, supervised the collection and processing of the data, developed the neurological insights, and led the writing of the manuscript.

### Conflict of Interest

The authors declare that the research was conducted in the absence of any commercial or financial relationships that could be construed as a potential conflict of interest.
